# Integrating network pharmacological and experimental models to investigate the therapeutic effects of baicalein in glaucoma

**DOI:** 10.1186/s13020-021-00537-9

**Published:** 2021-11-25

**Authors:** Jiawei Yang, Mingxu Zhang, Qiuyi Song, Siqi Li, Xiulan Zhao, Liping Kan, Siquan Zhu

**Affiliations:** 1 Eye School, Chengdu University of Traditional Chinese Medicine, 37 Shi Er Qiao Road, Chengdu, 610075 China; 2 Ineye Hospital of Chengdu University of TCM, Chengdu, China; 3 Department of Ophthalmology, Beijing Anzhen Hospital, Capital Medical University, Beijing, China

**Keywords:** Glaucoma, Baicalein, Network pharmacological, Experimental validation

## Abstract

**Background:**

Traditional Chinese medicine (TCM) has a long history of treating glaucoma with remarkable effects, but there is no clear conclusion on its mechanism.

**Methods:**

Network pharmacology and molecular docking were used to analyze the mechanism and targets of TCM in the treatment of glaucoma, and baicalein was used to treat chronic ocular hypertension animal models rats for observation.

**Results:**

The results of animal experiments showed that baicalein could significantly reduce intraocular pressure (IOP) in a rat model of chronic ocular hypertension and protect the structure of the retina and optic nerve, as shown by hematoxylin–eosin (H&E) staining and transmission electron microscopy (TEM). Reducing the apoptosis of retinal ganglion cells (RGCs) by upregulating the expression of the antiapoptotic protein BCL-2 is basically consistent with the results of molecular docking. In the network pharmacology analysis, many key proteins of biological pathways involved in the herbal therapeutic processes in glaucoma, such as threonine kinase 1 (AKT1, core protein of PI3K/AKT signaling), tumor protein p53 (TP53, a tumor suppressor gene coding tumor protein P53), signal transducer and activator of transcription 3 (STAT3, core protein of JAK/STAT signaling), interleukin 6 (IL-6) and interleukin 17 (IL-17, proinflammatory factors), were identified. Their interactions built complicated chain reactions in the process of glaucoma.

**Conclusion:**

By combining the analysis of network pharmacology and animal experimental results, baicalein could effectively improve the symptoms of glaucoma and reduce RGC apoptosis, suggesting that the potential mechanism of TCM in treating glaucoma is related to regulating inflammation and cellular immunity and reducing apoptosis.

## Introduction

Glaucoma, an optic neuropathy, has become a leading cause of irreversible vision loss due to its unpredictable morbidity and atypical initial symptoms, resulting in profound visual impairment worldwide. According to a report of the World Health Organization (WHO), approximately 76 million people (40–80 years old) suffer from vision loss caused by glaucoma, and glaucoma has become the fifth leading cause of vision impairment worldwide [1]. The etiology of glaucoma can be divided into primary, which is usually unknown, and secondary, which can be attributed to trauma, drugs, uveitis, and so on. However, it can also be classified as open-angle glaucoma (OAG) and angle-closure glaucoma (ACG) according to pathological changes in the anterior chamber angle (ACA). ACG always exhibits rapid visual impairment and drastic eye pain or headache. However, in contrast with ACG, OAG has chronic and nearly asymptomatic progression, allowing it to cause disastrous damage to retinal ganglion cells (RGCs) before the patient visits the hospital [2].

**Fig. 1:**
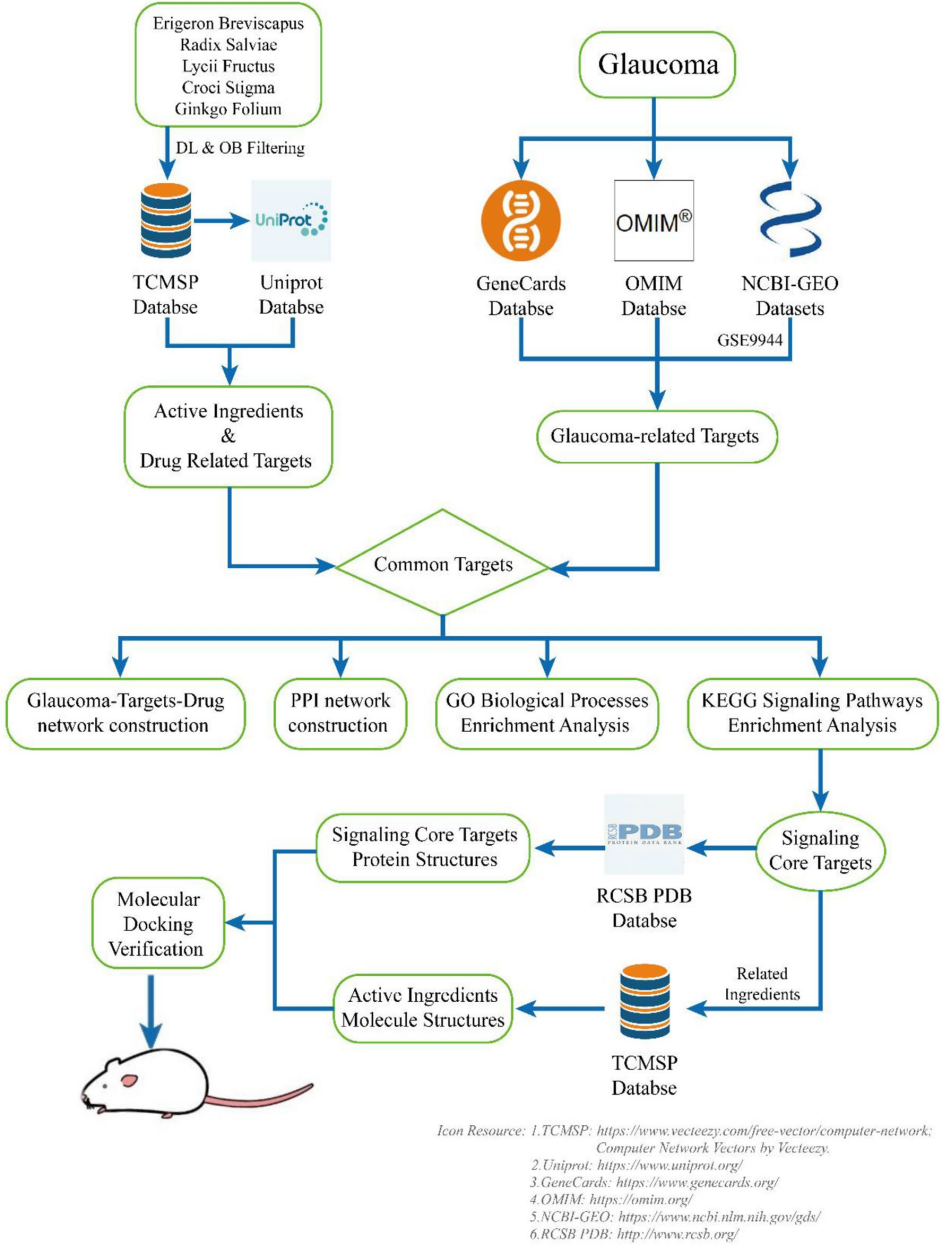
Schematic illustration of the network pharmacological analysis and molecular docking-based study

The risks of glaucoma include family inheritance, black race, senior age, use of corticosteroids and high IOP [2–4]. However, the management of IOP, including drugs, surgical treatments and prognostic estimates, is the only verified method that has been widely used in the clinic to slow disease progression [5–7]. Loss of RGCs is the critical pathological process of glaucoma [8] and is directly responsible for irreversible vision impairment. Due to the importance of RGCs in the pathology of glaucoma and the limits of current treatments, ophthalmological practitioners and researchers have paid more attention to the neuroprotection and regeneration of RGCs [9, 10]. TCM is widely used in the prevention and treatment of glaucoma in China, and some studies have found profound neuroprotective effects of TCMs [11]. *Erigeron breviscapus, Radix salviae, Lycii fructus, Croci stigma* and *Ginkgo folium* are 5 herbs that are commonly used in the treatment of glaucoma in China [12, 13]. However, due to the multicomponent and complex effects of TCMs, an appropriate method to elucidate the treatment mechanism of TCM has been lacking. Network pharmacology is a multiple and systemic research method to determine the treatment mechanism of drugs based on systems pharmacology and network biology, overcoming the obstacles noted above [14]. In the present study, we constructed glaucoma-target drug and protein–protein interaction (PPI) networks based on herbal active ingredients and drug-disease common targets acquired from different databases and performed Gene Ontology (GO) and Kyoto Encyclopedia of Genes and Genomes (KEGG) pathway enrichment analyses. Finally, we performed molecular docking verification of the herbal active ingredients and their target proteins, which play important roles in signaling pathways related to glaucoma, allowing us to understand the possible connective patterns and binding energies of active ingredients with target proteins. We constructed an animal model of chronic ocular hypertension (COH) and administered baicalein to the model group for observation. We exhibit the work procedures of our research as a schematic illustration to facilitate understanding in Fig. 1.

## Methods

### Herbal data collection

The active ingredients of *Erigeron breviscapus, Radix salviae, Lycii fructus, Croci stigma* and *Ginkgo folium* were acquired from the Traditional Chinese Medicine Systems Pharmacology (TCMSP) Database (http://tcmspw.com), which focuses on TCM [15], under the screening conditions of oral bioavailability (OB) ≥ 30% and drug-likeness (DL) ≥ 0.18. We also used TCMSP to obtain drug-related targets (DRTs) and then acquired their gene symbols from the UniProt Database (https://www.uniprot.org).

### Acquisition of glaucoma-related targets

Disease target information was collected from 3 databases: the Online Mendelian Inheritance in Man Database (OMIM, https://omim.org), GeneCards Database (https://www.genecards.org) and GEO Datasets of NCBI (https://www.ncbi.nlm.nih.gov/gds) [16]. We searched the key term “glaucoma” in OMIM and GeneCards to obtain targets and in GEO to obtain the gene chip data of GSE9944 and the platform files of GPL571 and GPL8300. The screening of differential gene expression was performed using the limma package for R software (version 3.6.0) with the filters of *P* > 0.05 and |log_2_ fold change (FC)|> 0.5. Finally, we summarized the targets and differentially expressed genes above into known glaucoma-related targets (GRTs).

### Construction of glaucoma-targets-drug and protein–protein interaction (PPI) networks

The drug-disease common targets were collected via R software (version 3.6.0) and are shown as a Venn diagram. Then, we used Cytoscape software (version 3.7.2) to construct and visualize the Glaucoma-Targets-Drug network. Under the screening conditions of “Organism = Homo sapiens” and “High Confidence (> 0.9)”, the common targets were also used to build the PPI network using the STRING Platform (version 11.0). The nodes and edges between the nodes consisted of the network above, in which the degree of the node is the number of edges connected to the node, and a higher degree indicates greater importance of the node.

### Gene ontology (GO) biological processes enrichment analysis

To explore the functional processes of common drug-disease targets, we performed GO biological process enrichment analysis on these targets in the R/Bioconductor environment (http://www.bioconductor.org/). Based on the gene ratio, we show the top 20 processes (*P* < 0.05) as a histogram and bubble chart.

### Kyoto Encyclopedia of genes and genomes (KEGG) pathway enrichment analysis and construction of a functional pathway network

To clarify the potential molecular mechanisms, KEGG pathway enrichment analysis was performed by Metascape Platform software (https://metascape.org/), and we also created a heatmap of the top 20 KEGG pathways. Then, 8 pathways highly related to glaucoma were selected to construct the functional pathway network, which also involved related targets and active ingredients. The functional pathway network was constructed using Cytoscape software.

### Molecular docking study

We performed molecular docking to verify the connective validity of active ingredients and core targets using AutoDock Vina software (version 1.1.2) [17]. The 3D structures of active ingredients and target proteins were obtained from the TCMSP database and the RCSB PDB database (http://www.rcsb.org/), respectively. The proteins were dehydrated and hydrogenated, and a grid box was set via AutoDockTools software (version 1.5.6). To determine exhaustive docking patterns, the docking of ingredients and targets was performed in a flexible and unrestrained manner, allowing the ligand to move through the entire volume of the grid box. After the steps above, the docking results were visualized using PyMOL software (https://www.pymol.org).

### Animal and environmental conditions

Male Sprague–Dawley (SD) rats (200 ± 20 g) from the Laboratory Animal Centre, Chengdu University of Traditional Chinese Medicine, were used, and the experiment was approved by the Committee of Scientific Research and the Committee of Animal Care of Chengdu University of Traditional Chinese Medicine (permission number: 2020–12). Animals were adaptively fed for one week at a cozy temperature of 23 ± 2 ℃ and temperate humidity of 40–60%. We abided by guidelines about animal use from the Association for Research in Vision and Ophthalmology (ARVO) throughout the study.

### Chemicals and antibodies

Baicalein (S25956), sodium carboxymethyl cellulose (CMC-Na, S14017) and balanced salt solution were purchased from Shanghai Yuanye Bio-Technology Co., Ltd. (Shanghai, China). A TdT-mediated dUTP nick-end labeling (TUNEL) staining kit (11684795910) was purchased from Roche Group (Basel, Switzerland). BCL-2 (bs-20351R) was procured from Bioss Co., Ltd. (Beijing, China). FITC-conjugated goat anti-rabbit IgG (GB22303) and FAS eyeball fixative (G1109) were provided by Servicebio (Wuhan, China). DAPI (ZLI-9557) and citrate repair solution (ZLI-9065) were obtained from Zsbio Co., Ltd. (Beijing, China). Newborn calf serum (22012-8612) was obtained from Tianhang Co., Ltd. (Hangzhou, China).

### Animal modeling, grouping and treatment

A total of 20 SD rats were anesthetized by intraperitoneal injection of 2% pentobarbital sodium solution at a dose of 50 mg/kg weight and then underwent episcleral vein cauterization (EVC) surgery in the right eye by the same skillful surgeon. EVC surgery complied with the procedures designed by Shareef [18]: three small incisions of the conjunctiva in the right eye were created to expose the episcleral veins, and then three vein stems were cauterized using a cautery pen. The left eyes of the sham-operated group rats received a sham procedure that only opened the conjunctiva to expose the episcleral veins and did not cauterize them. Random grouping after the modeling was performed as follows: 10 rats underwent sham operations on the left eyes as a control group (CON) as with as right eye modeling as COH (intact left eyes were used as self-contrast), and 10 rats in the BAL (right-eye modeling). The intragastric administration of drugs began one day after modeling surgery, until the end of the experiment 28 days later: the groups above received suspensions of baicalein (200 mg/kg weight) and 0.5% CMC-Na solution. Gavage was performed in the morning from 10:00–12:00 to avoid possible differences between days.

### IOP measurement

The IOP assay was performed preoperatively (day surgery), the first day after surgery (before drug administration), the third day of surgery, the seventh day after surgery, the fourteenth day after surgery, and the twenty-first day after surgery by a TONOLAB tonometer TV02 (Icare, Finland). The measurements of IOP were performed at least three times on each eye, and the integer nearest to the mean value of the measurement results above was recorded as the final IOP of the eye. During the measurements, the animal was in a fully conscious state. According to the IOP results of the first day after surgery (before drug administration), we excluded with IOP (operated eye) < 22 mmHg from the subsequent experiments.

### Hematoxylin and eosin stain

H&E staining was performed to quantify the thickness of the ganglion cell complex (GCC) and changes in retinal structure. After 28 days, rats were sacrificed by anesthesia, and their eyeballs were removed. Ocular tissues were fixed by formaldehyde, acetic acid, and saline (FAS) eyeball fixative, dehydrated with gradient concentrations of ethanol, and then sliced into 7 μm-thick slides with a Rotary Slicer-RM2016 (Leica, Germany). Slides stained with H&E were sealed with neutral balsam, with 3 samples per group and 3 slices on average. The overall GCC thicknesses (from the ganglion cell layer (GCL), to the nerve fiber layer (NFL)) were measured. All measurements were carried out at distances of 1.0–1.5 mm (central) retina from optic disc with ImageJ software (Bethesda, MD, USA).

### Terminal deoxynucleotidyl-transferase (TdT)-mediated dUTP nick end-labeling (TUNEL) assay

Rats were sacrificed after 28 days, and three eyes were removed from each group. TUNEL assay was performed to quantify RGC apoptosis in the retina. TUNEL staining was processed by an In Situ Cell Death Detection Kit, Fluorescein (Roche Group, Switzerland). After dewaxing, the slides were repaired with citric acid microwave for 8 min, washed with phosphate buffer saline (PBS) three times, 5 min each time. Prepared with fluorescent TUNEL incubation solution in the dark (A:B = 1:30) and incubated for 1 h at the temperature of 37 °C, washed three times with PBS, and stained with DAPI for 15 min, followed by washing with PBS. The slides of the TUNEL stain were sealed by gelatin glycerin. The images of the slides above were collected and analyzed by Pannoramic 250 Flash (Danjier, Jinan, China).

### Immunofluorescence staining of the retina

After the experiment, the rats were sacrificed, and the eyeballs were removed. Ocular tissues were fixed by formaldehyde, acetic acid, and saline (FAS) eyeball fixative, dehydrated in sucrose, and then sliced into 7 μm-thick slides with a Rotary Slicer-RM2016 (Leica, Germany). After dewaxing, the slides were immersed in 0.01 M citrate buffer (pH 6.0), washed three times with PBS for 5 min each time, and blocked at room temperature for 30 min in newborn calf serum. Primary antibody was added (BCL-2 1:100) dropwise, followed by refrigeration overnight at 4 °C, washing three times with PBS for 5 min each time, addition of the secondary antibody dropwise 37 °C for 30 min, washing with PBS the same as before, addition of DAPI and incubation at room temperature for 10 min, washing with PBS and sealing with anti-fluorescence attenuation reagent. The images of the slides above were collected and analyzed by a Pannoramic 250 Flash (Danjier, Jinan, China).

### Transmission electron microscopy for optic nerve

Rats were sacrificed after 28 days, and three optic nerves were removed from each group. The optic nerve samples were fixed in 3% glutaraldehyde and postfixed in 1% osmium tetroxide (Leica Company, Germany). After dehydration by gradient acetone dehydration, the optic nerve samples were permeated in Epon 812 epoxy resin (Beijing Keyi, Beijing, China). Then, the permeated samples were embedded in flat molds and heating polymerized for embedded blocks. Ultrathin sections (50 nm) were created with an ultramicrotome and double-stained with Reynolds’s lead citrate and 0.5% aqueous uranyl acetate (Beijing Keyi, Beijing, China). The microscopic examination was performed with a JEM-1400PLUS TEM (JEOL, Tokyo, Japan), and the images were analyzed with OlyVIA software (version 2.8).

### Statistical analyses

SPSS software (Version 26.0) was used for statistical analysis, and GraphPad Prism software (Version 7) was used for plotting. All of the images were measured by ImageJ software (Bethesda, MD, USA). Data are expressed as the mean ± standard deviation (SD). Measurement data conformed to a normal distribution and were compared by the independent sample t test analysis for two groups. One-way ANOVA was used for multigroup analysis; the measurement data did not conform to a normal distribution, and the nonparametric rank sum test was used. *P* < 0.05 was considered statistically significant.

## Results

### Screening of active ingredients and drug related targets

Under the screening conditions of OB ≥ 30% and DL ≥ 0.18, we selected 143 active ingredients from the total ingredients of 5 traditional Chinese medicines (*Erigeron breviscapus, Radix salviae, Lycii fructus, Croci stigma* and *Ginkgo folium*) and obtained 226 DRTs from the TCMSP database. We list some active ingredients in Table 1.

**Table 1:** Active ingredients (part) of TCMs

MolID	Name	MolID	Name
MOL000098	Quercetin	MOL007093	Dan-shexinkum d
MOL000006	Luteolin	MOL007963	1-Hydroxy-2,3,5-trimethoxy-xanthone
MOL000422	Kaempferol	MOL007119	Miltionone I
MOL002714	Baicalein	MOL007111	Isotanshinone II
MOL007154	Tanshinone iia	MOL000358	Beta-sitosterol
MOL000392	Formononetin	MOL007049	4-Methylenemiltirone
MOL000354	Isorhamnetin	MOL007124	Neocryptotanshinone ii
MOL000449	Stigmasterol	MOL007108	Isocryptotanshi-none
MOL008400	Glycitein	MOL007088	Cryptotanshinone
MOL007100	Dihydrotanshinlactone	MOL007041	2-Isopropyl-8-methylphenanthrene-3,4-dione

### Glaucoma-Related target acquirements and drug-glaucoma common target filtering

**Fig. 2:**
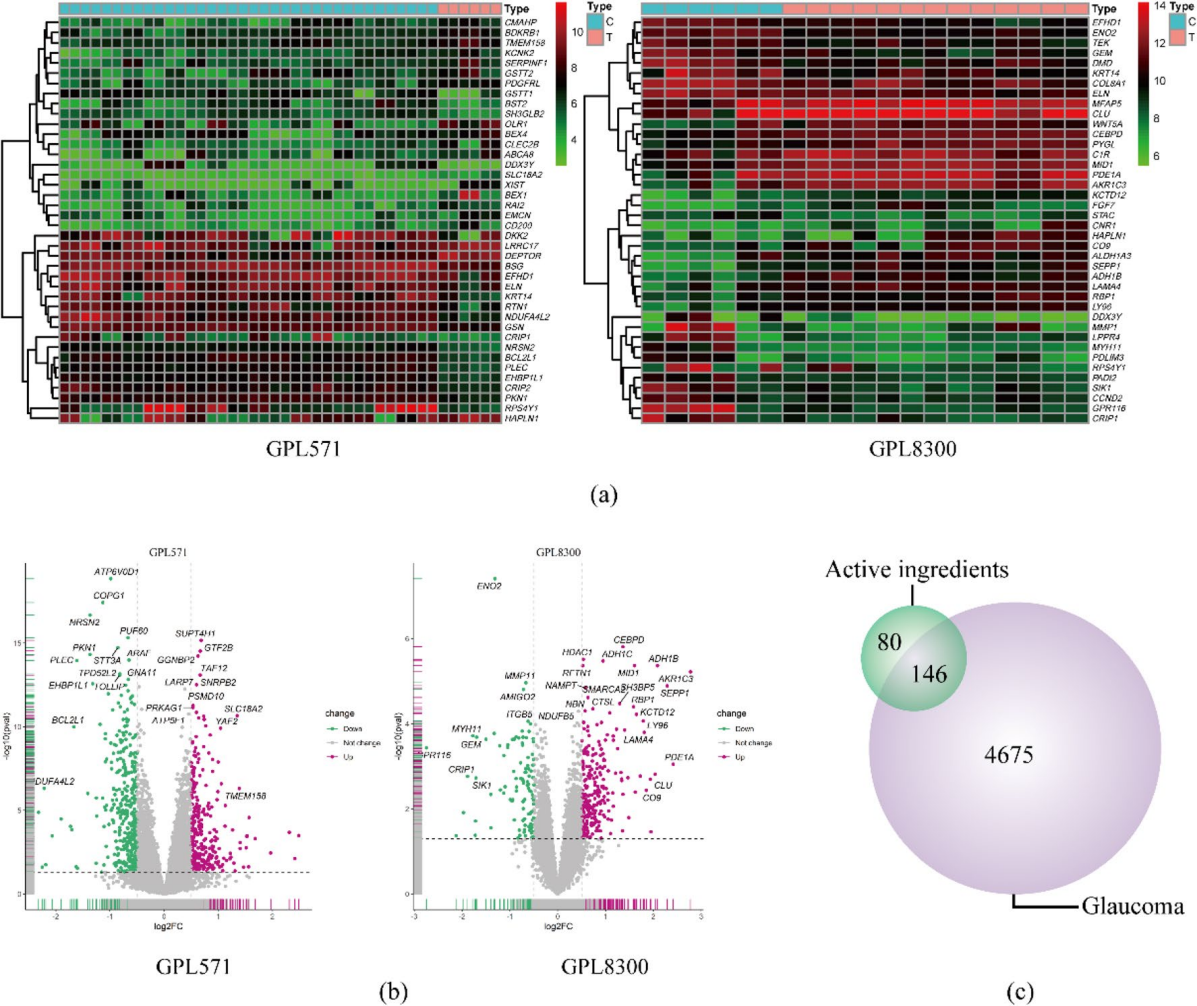
Glaucoma-related differential gene analysis and drug-disease common target collection. **a** Heatmap of differential gene analysis of GSE9944. **b** Volcano plot of differential gene analysis of GSE9944. **c** Drug-disease common target collection

We acquired 53 and 4130 glaucoma-related targets from the OMIM and GeneCards databases, respectively. We analyzed the gene chip GSE9944 and related platform files GPL571 and GPL8300 obtained from GEO datasets by searching the key term “glaucoma”, and the results provided 940 differential genes (DEGs), which were exhibited as heatmaps and volcano plots using R software (Fig. 2a, b). According to the DEG analysis, there were 47 common DEGs in volcano plots of GPL571 and GPL8300. Summarizing the targets and differentially expressed genes above, we obtained 4821 GRTs in total. A total of 146 drug-disease common targets (Fig. 2c) were collected by the intersection of GRTs and DRTs performed using R software.

### Glaucoma-targets-drug network analysis

**Fig. 3:**
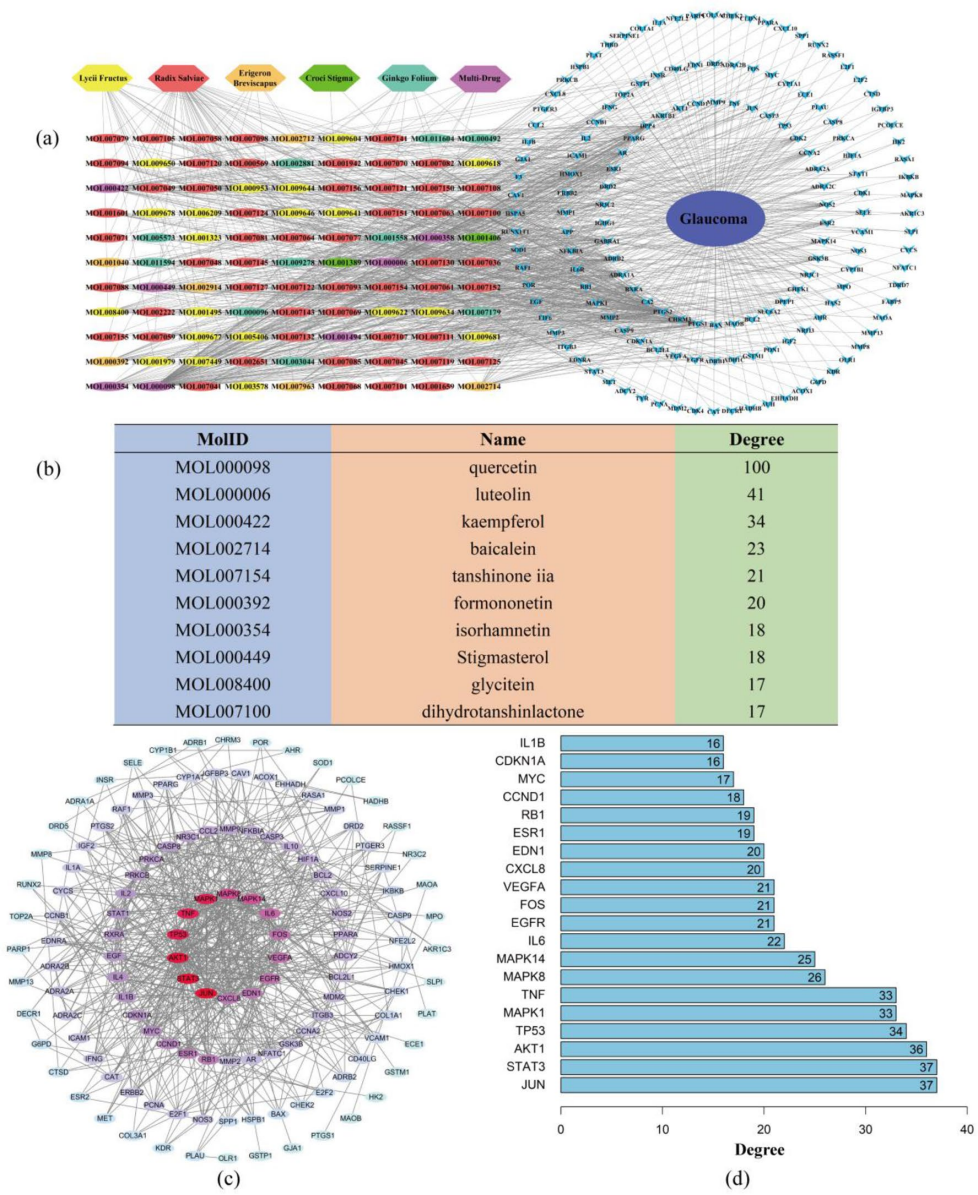
Glaucoma-target-drug network and common target PPI network analysis. **a** Glaucoma-targets-drug network. **b** Degree of active ingredients of TCMs (top 10). **c** PPI network of common targets (degrees range from high to low as red to blue). **d** Degree (number of node-connected edges) of common targets (top 20) in PPI network

Using Cytoscape, we constructed a glaucoma-target drug network based on 146 common targets and 99 glaucoma-related active ingredients of 5 herbs (Fig. 3a). The active ingredients are shown as the MolIDs acquired from TCMSP and were stained by different colors to distinguish the herbal origins. Among these glaucoma-related active ingredients, 2, 6, 10, 20, and 54 belong to *Croci stigma, Erigeron breviscapus, Ginkgo folium, Lycii fructus* and *Radix salviae*, respectively, but there are 7 ingredients that are contained in more than 2 herbs. The ingredients and targets were laid out as 2 and 3 concentric circles, respectively, according to their degrees (inner > outer). The top 10 active ingredients according to degree are listed in Fig. 3b.

### Drug-Glaucoma common targets protein–protein interaction (PPI) network analysis

As shown in Fig. 3c, we constructed the PPI network based on 146 common targets on the String Platform. The nodes in the PPI network represent different target proteins, and they are connected by edges. We excluded disconnected nodes and nodes with low interaction scores (< 0.9) from the PPI network and finally acquired 146 nodes and 626 edges. According to the degree (number of node-connected edges) of nodes, we exhibited the top 20 targets as a histogram (Fig. 3d). The proteins are shown as gene symbols, and some of them, such as STAT3, AKT1, TP53, and TNF, are involved in the pathogenesis and development of glaucoma.

### GO enrichment analysis of drug-glaucoma common targets

**Fig. 4:**
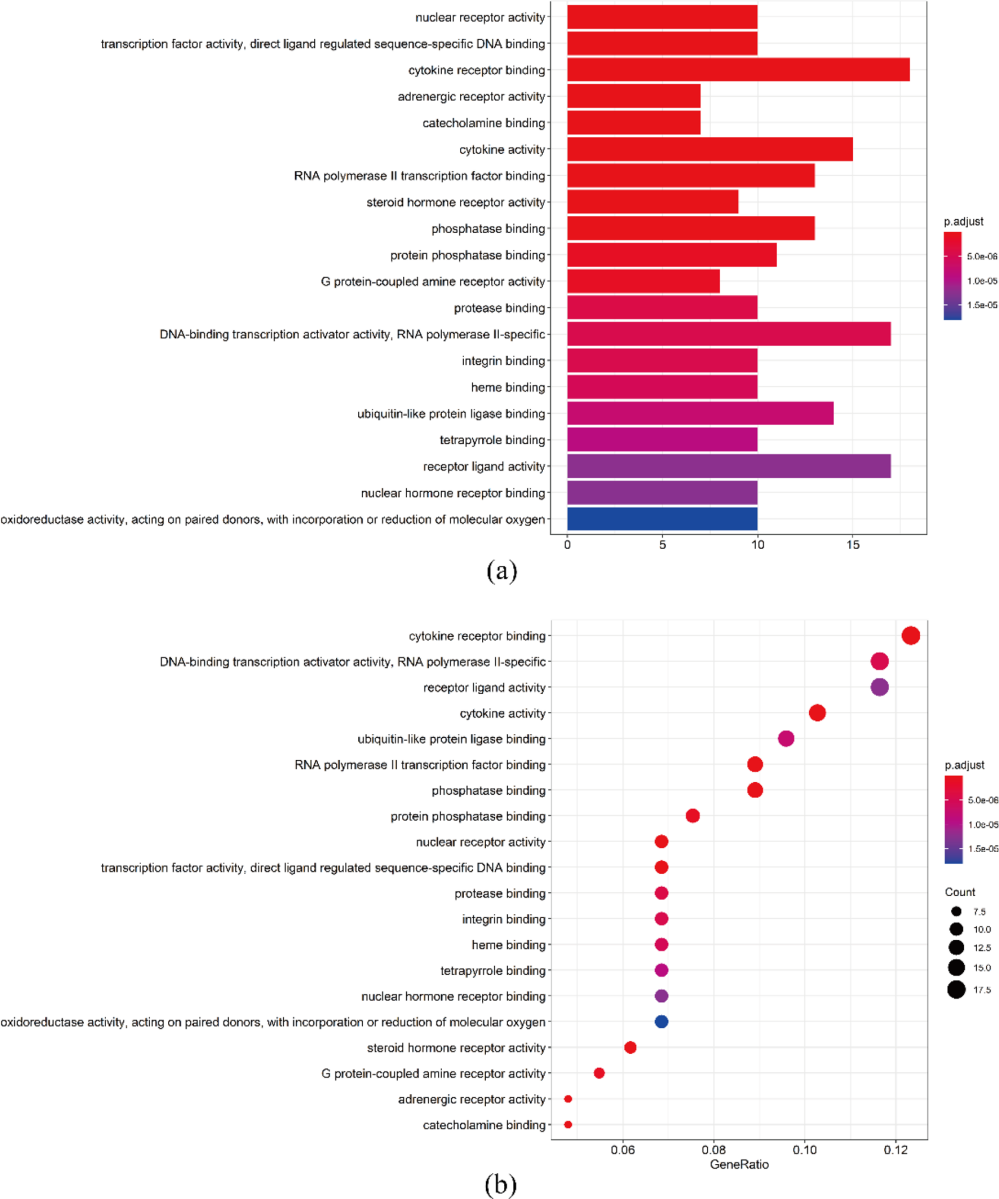
GO-enrichment analysis of common targets. **a** Histogram of the top 20 biological processes according to gene counts. **b** Bubble chart of the top 20 biological processes according to gene counts

After the PPI analysis, we performed GO enrichment analysis with R software to clarify the biological processes that were involved and how they acted in the etiology of glaucoma, as well as the herbal therapeutic effects. There were 148 biological processes with *P* < 0.05, and the gene count determined the importance of the process. We built a histogram and bubble chart (Fig. 4a, b) of biological processes (top 20) according to gene counts (x axis), and the gradient-colored bars/bubbles changed with the P values of related processes. The biological processes above are involved in multiple functions of cellular metabolism, signaling communications, gene expression and so on, such as cytokine receptor binding, nuclear receptor activity, transcription activator and RNA polymerase activity.

### KEGG enrichment analysis of drug-glaucoma common targets

**Fig. 5:**
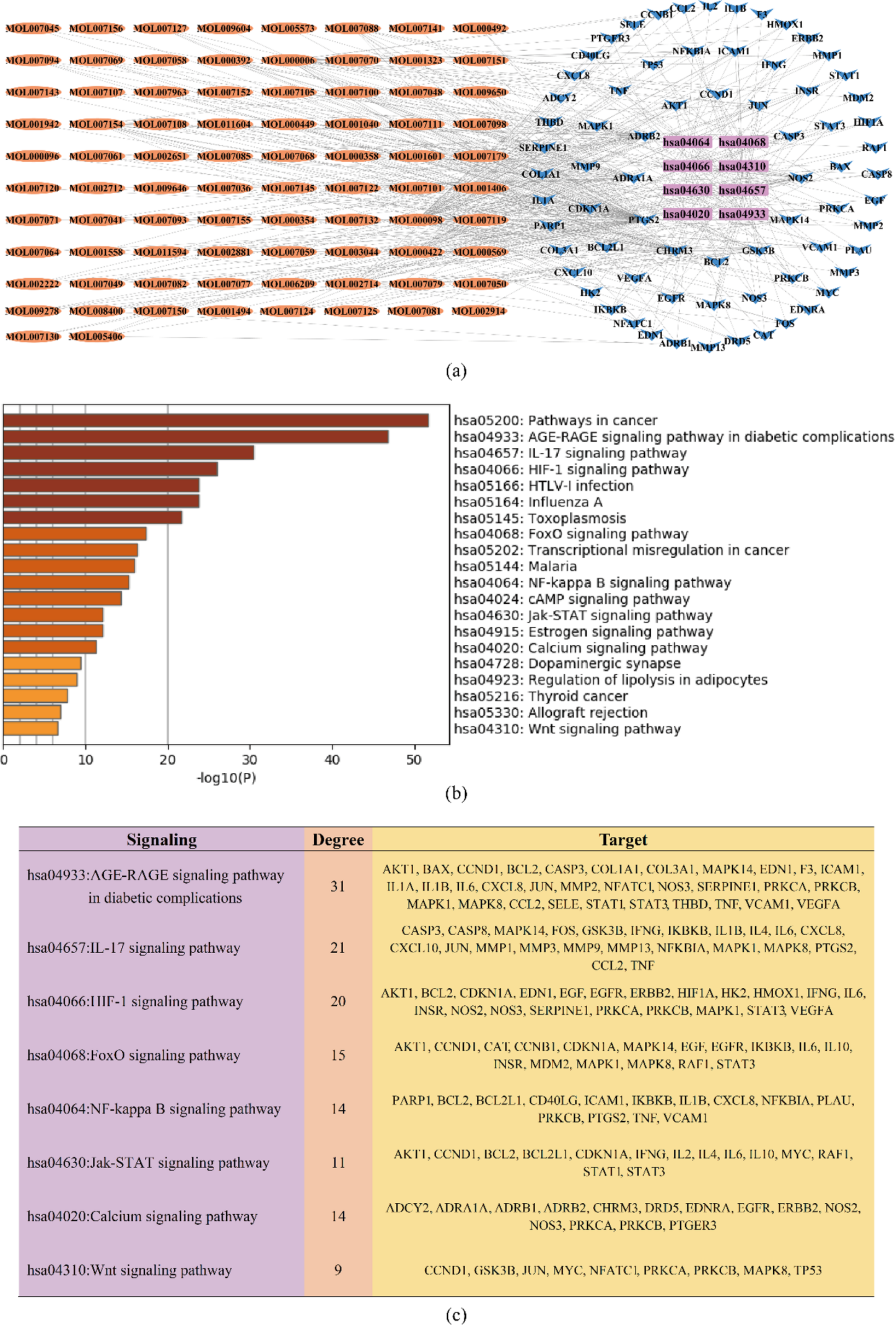
KEGG enrichment analysis of common targets. **a** Functional pathway network based on KEGG signaling enrichment analysis. **b** Histogram of the top 20 signaling pathways according to -log_10_(P). **c** Important glaucoma-related signaling pathways and their target proteins

We acquired 178 signaling pathways (*P* < 0.05) from the KEGG signaling pathway enrichment analysis performed by the Metascape platform. The top 20 biological signaling pathways are shown in Fig. 5b according to − log_10_(P). Then, we chose some signaling pathways highly related to glaucoma, the common targets involved in these pathways and the active ingredients of herbs to build the functional pathway network (Fig. 5a), which contained 8 pathways, 71 common targets and 82 active ingredients (shown as MolID). The 8 signaling pathways of the network included the AGE-RAGE signaling pathway, IL-17 signaling pathway, HIF-1 signaling pathway, FoxO signaling pathway, NF-κB signaling pathway, Jak-STAT signaling pathway, calcium signaling pathway and Wnt signaling pathway (Fig. 5c).

### Molecular docking verification of core targets and active ingredients

**Fig. 6:**
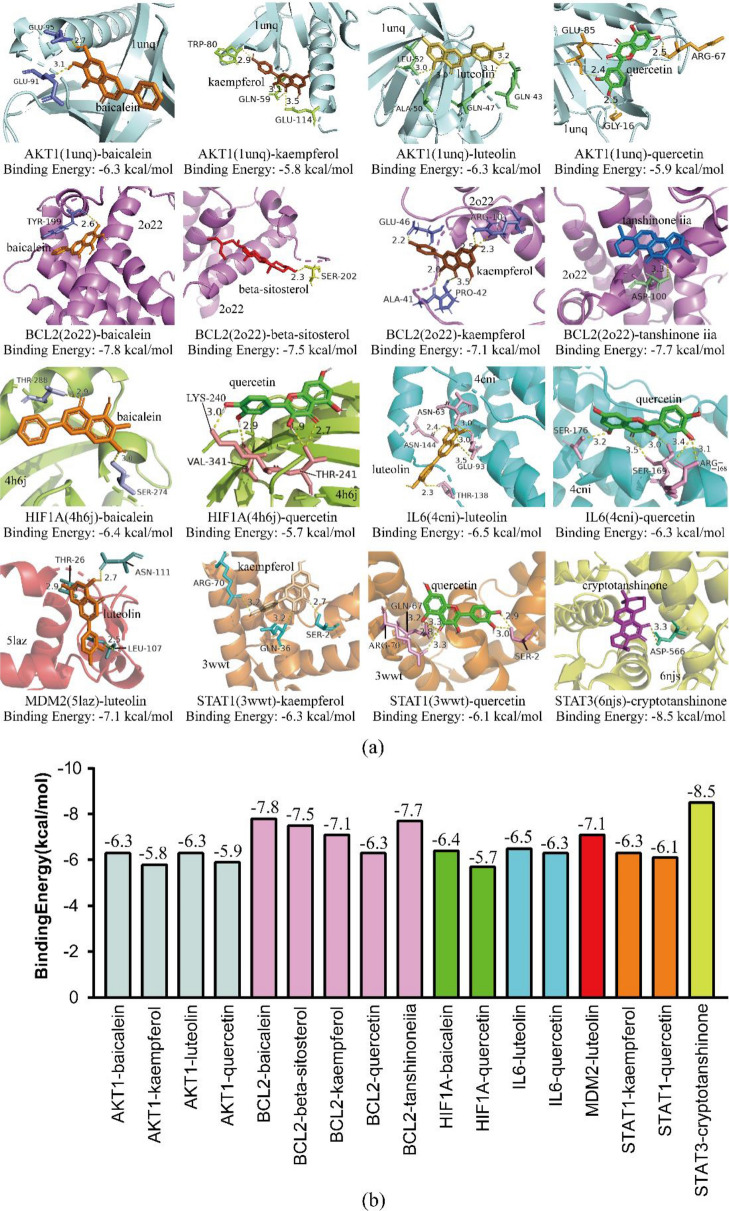
Molecular docking analysis. **a** Docking patterns of core proteins in glaucoma-related signaling pathways. **b** Binding energy of docking results

Based on the results of KEGG signaling pathway enrichment analysis, we performed molecular docking on the core target proteins of the important signaling pathways, including AKT1 (1unq), BCL2 (2o22), HIF1A (4h6j), IL6 (4cni), MDM2 (5laz), STAT1 (3wwt) and STAT3 (6njs). We obtained the active ingredients related to these core targets from the analysis of the glaucoma-target-drug network, including quercetin (MOL000098), baicalein (MOL002714), luteolin (MOL000006), kaempferol (MOL000422), beta-sitosterol (MOL000358), tanshinone iia (MOL007154) and cryptotanshinone (MOL007088). The 3D structures of ingredient molecules and core target proteins were obtained from the TCMSP and RCSB PDB databases, respectively. After dehydration and hydrogenation, we performed docking with AutoDockTools and AutoDock Vina software. The absolute value of the binding energy is positively correlated with the connective stability of the ingredient molecule and target protein, and we show the most stable connective patterns in Fig. 6a. Details of the binding energies are listed in Fig. 6b. According to the network analysis and molecular docking simulation, the candidate drug for the in vivo experiment was baicalein, which targeted multiple glaucoma-related targets and had a powerful binding pattern with the apoptosis-related target BCL-2.

### IOP change

**Fig. 7:**
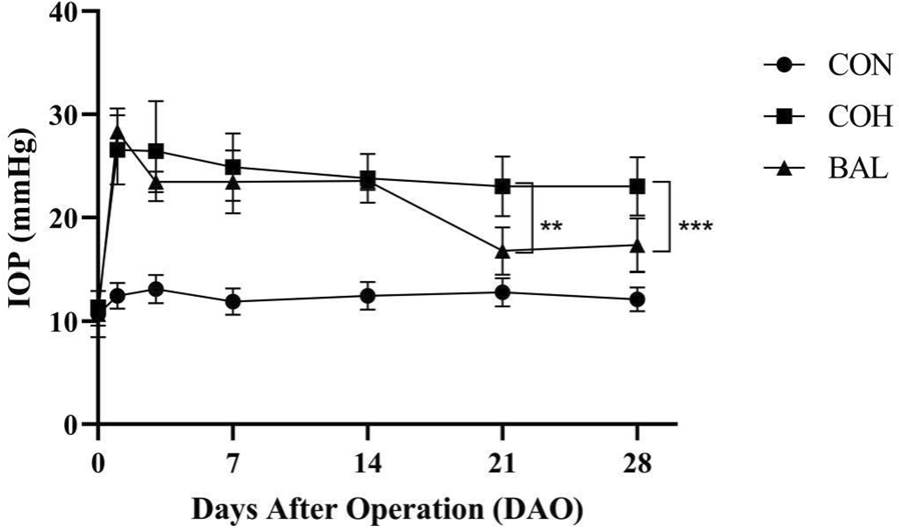
Effects of BAL on IOP change during the experiment (mmHg). **P < 0.01, ***P < 0.001

IOP was continuously monitored in each group before modeling and at 1, 3, 7, 14, 21, and 28 days after the operation. Glaucoma significantly lead to IOP increase of rats, compare preoperation (COH:11.33 ± 0.71 mmHg, BAL:10.67 ± 2.24 mmHg) with one day after surgery, the COH and BAL group IOP increased to 26.56 ± 3.3 mmHg and 28.33 ± 2.2 mmHg, respectively (*P* < 0.0001). As shown in Table 2. However, compare with the COH group (21 days:23.00 ± 2.92 mmHg, 28 days:23.00 ± 2.8 mmHg), BAL group IOP decreased to 16.78 ± 2.28 mmHg, 17.33 ± 2.6 mmHg at 21 days, 28 days after surgery, respectively (*P* < 0.01) (Fig. 7).

**Table 2:** IOP after surgery (mmHg)

DAO	Preoperation	1	3	7	14	21	28
CON	10.67 ± 1.12	12.44 ± 1.2	13.11 ± 1.36	11.89 ± 1.27	12.44 ± 1.33	12.78 ± 1.39	12.11 ± 1.17
COH	11.33 ± 0.71	26.56 ± 3.3^▲^*	26.44 ± 4.85	24.89 ± 3.26	23.78 ± 2.39	23.00 ± 2.92*	23.00 ± 2.8*
BAL	10.67 ± 2.24	28.33 ± 2.2^▲^*	23.44 ± 1.01	23.44 ± 3.05	23.56 ± 0.88	16.78 ± 2.28*^#^	17.33 ± 2.6*^#^

All data are shown as mean ± SD, n = 10. Comparisons before and after surgery at different times ^▲^*P* < 0.0001, comparisons at 1/21/28 days after surgery with CON group *P < 0.01, comparisons at 21/28 days after surgery with COH group ^#^*P* < 0.01

### The effect of BAL on the thickness of GCC stained by HE

**Fig. 8:**
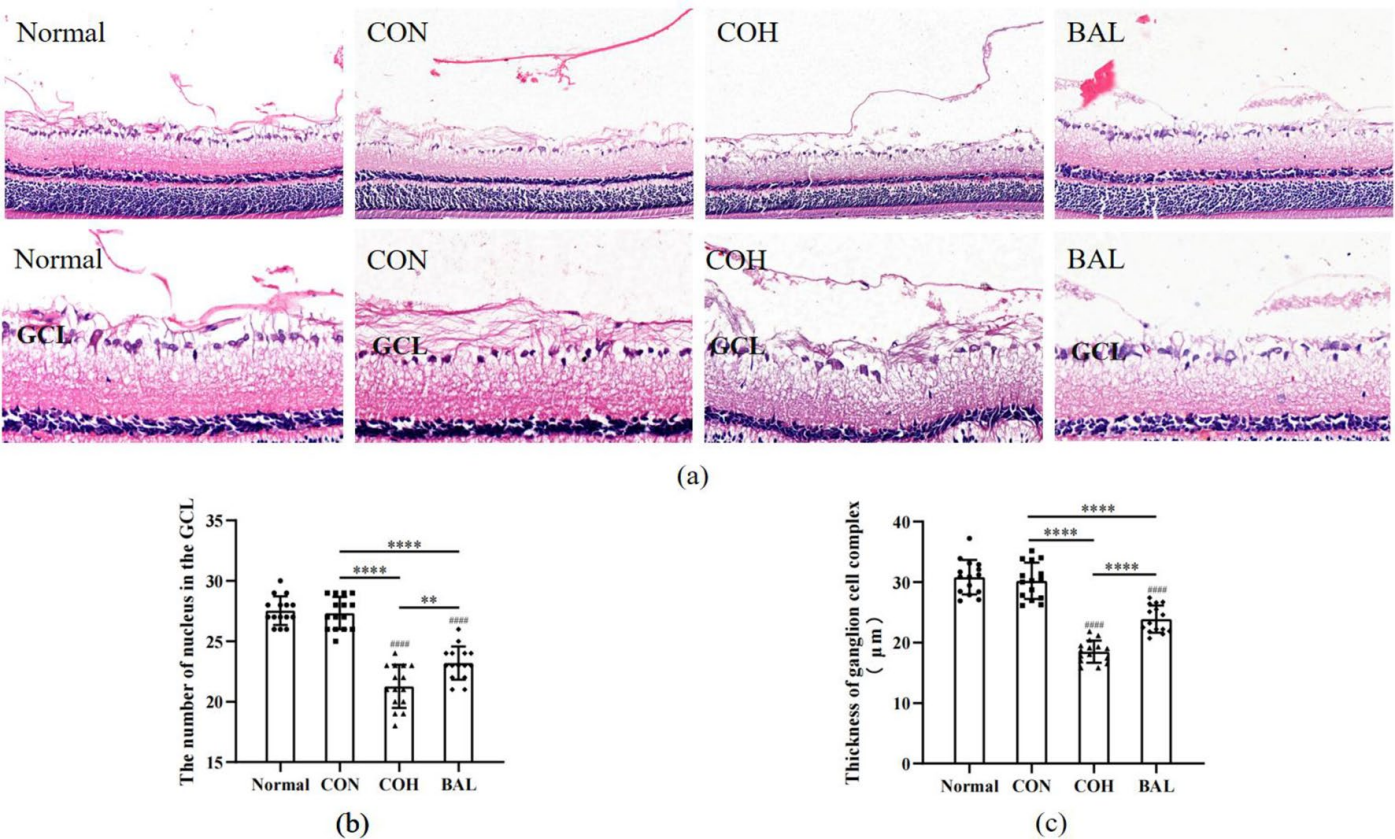
Baicalein and 0.5% CMC-Na solution were administrated consecutively at the dose of 200 mg/kg once per day for 28 days. Rats were sacrificed on the 29th day after model surgery. **a** Effects of BAL on histological changes of retina, all tissues were observed under a light microscope (bar = 20 μm; 10 μm), and representative pictures are shown. Normal showed normal retinal tissue. CON, control group showed normal well-arranged retinal layers. COH, chronic ocular hypertension group showed local GCC thickness decreased. BAL, baicalein group showed no obvious abnormalities in the arrangement of the retinal layers. **b** The number of nucleus (amacrine cells and RGC) in the GCL in rats. **c** Thickness of ganglion cell complex in retinal tissues in rats. All data are shown as mean ± SD, n = 3. **P < 0.01, ****P < 0.0001. ^####^*P* < 0.0001, compared with Normal

At the end of the experiment, rats were sacrificed and the eyeballs were removed for histological analysis. We observed morphological and structural changes in ganglion cells and nerve fibers using H&E staining (Fig. 8a). Normal represents normal retinal tissue. In CON, showed normal well-arranged retinal layers, normal morphology and structure of ganglion cells and the nerve fiber layer. In COH, H&E staining revealed that the structure of the NFL was disordered, the number of nucleus in the GCL decreased, the thickness of the GCC was obviously decreased (*P* < 0.0001) (Fig. 8). However, compared with COH, the thickness of GCC decreased mediated by chronic ocular hypertension induction was markedly ameliorated by BAL (*P* = 0.0064) (Fig. 8c), the number of nucleus in the GCL increased (*P* < 0.01) (Fig. 8b), the structural of retina and RGCs were no obvious abnormally. These results indicate that BAL has a protective effect against glaucoma damage in rats.

### The effect of BAL on TUNEL apoptosis and immunofluorescence staining assay of retinas

**Fig. 9:**
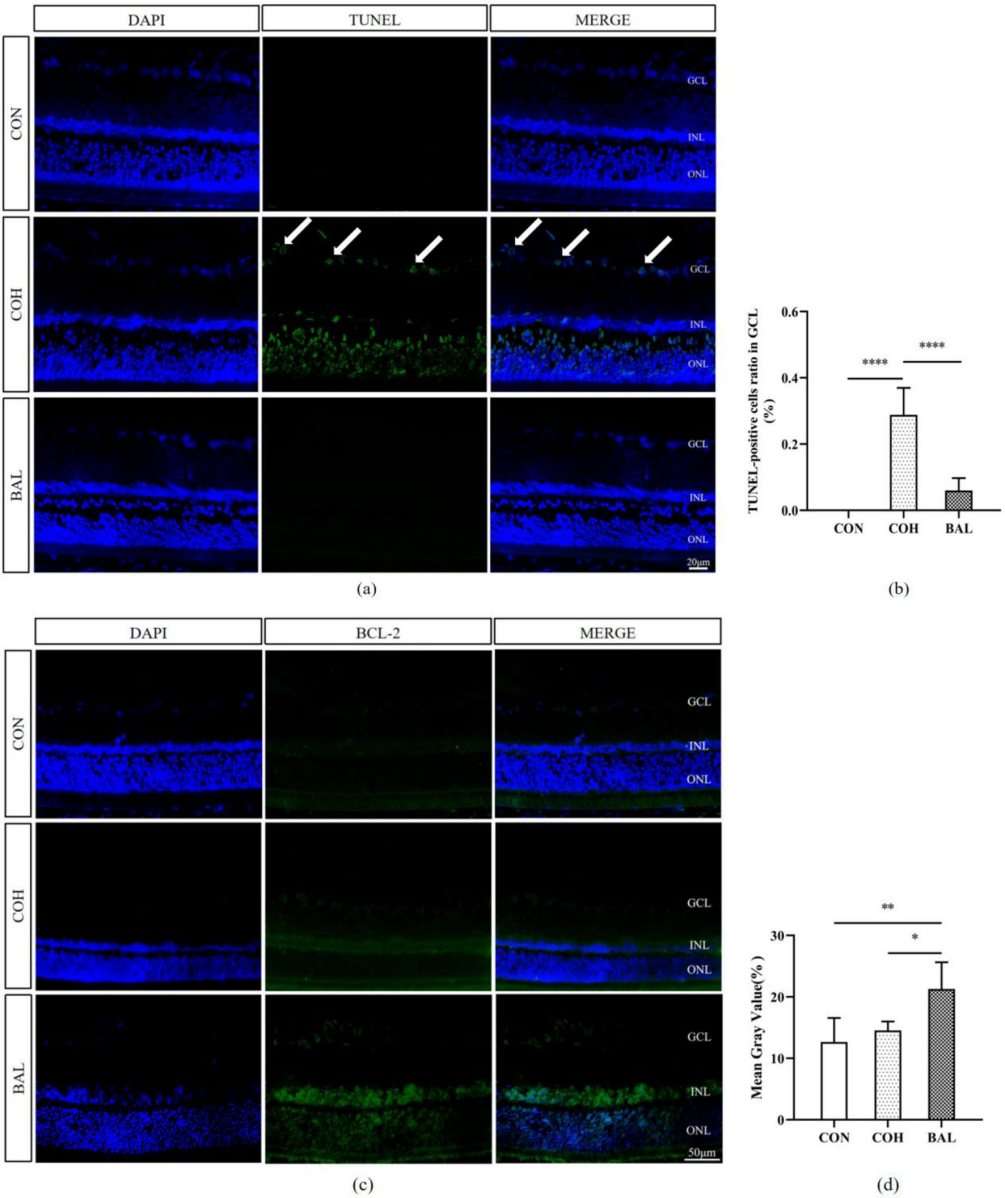
**a** Immunofluorescent staining of TUNEL (× 400) in CON, COH and BAL group. Apoptotic cells are stained by green color, and the nucleus is stained by blue. The apoptosis-positive staining assembled in the GCL (arrow). Scale bar = 20 μm. **b** The apoptosis rate in GCL (TUNEL/DAPI) comparison among these three groups. **c** Immunofluorescent staining expression of BCL-2 (× 400) in CON, COH and BAL group. Scale bar = 50 μm. **d** Quantitative analysis of BCL-2 fluorescent positive protein expression. All data are shown as mean ± SD, n = 3. *P < 0.05, **P < 0.01, ****P < 0.0001

To further investigate the protective effects of BAL on glaucoma damage, particularly RGC apoptosis (a main mechanism of glaucoma), we evaluated the protein (Fig. 9a) expression of apoptosis-regulating factors by TUNEL and immunofluorescent staining (Fig. 9b). No significant TUNEL staining-positive cells were found in each field of CON. Significant TUNEL-positive cells were observed in the sections of COH (*P* < 0.0001) (Fig. 9a, b), and apoptosis-positive staining mainly assembled in the GCL and ONL. In BAL, no significant TUNEL-positive cells were found in GCL. Only a small amount of BCL-2 was expressed in the retina in CON, compared with CON, the COH and BAL group were increased. The BCL-2 expression was significantly higher than CON group in BAL (*P* < 0.01), but there was no significantly increased between COH and CON group (*P* > 0.05) (Fig. 9d).

### The effect of BAL on ultrastructural changes in the optic nerve

**Fig. 10:**
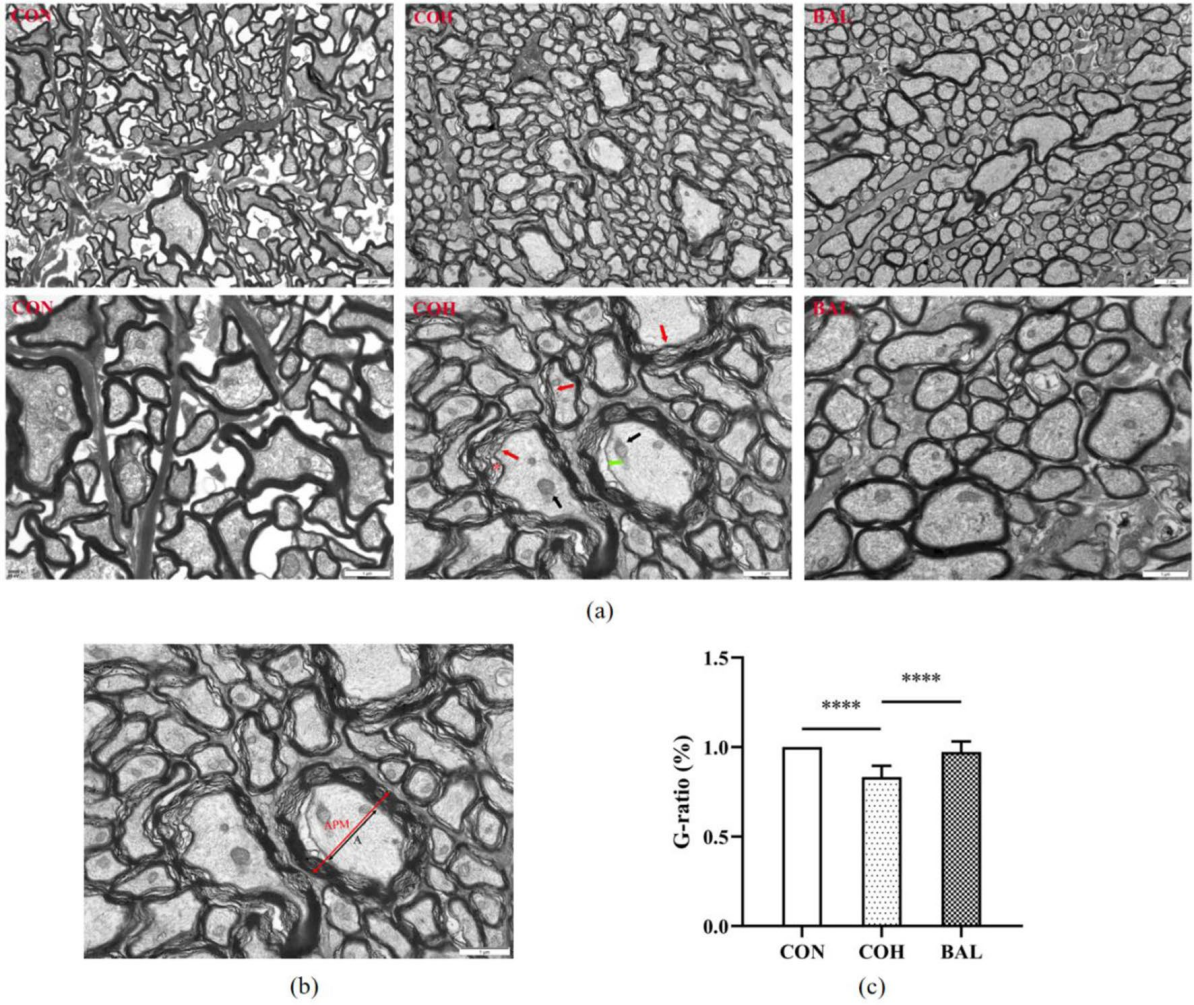
**a** Representative TEM images showing ultrastructural changes in optic nerve tissue in different groups (bar = 2 μm; 1 μm). CON showed healthy optic nerve tissue. COH showed a small number of myelinated axons swollen (green arrow), thickness of myelin sheath increased, separation (red arrow) and vacuolation (*) of myelin sheath, mild swelling of mitochondria (black arrow). BAL showed partial myelinated axons diameter increasing, but tightly arranged. **b** Axon only (black, bar = 1 μm) and axon plus myelin (red, bar = 1 μm) diameter of each group were measured under high power (× 20,000). **c** Quantitative analysis of G-ratio among the three groups. n = 3. ****P < 0.0001

CON group showed a normal optic nerve ultrastructural, axons and myelin sheaths are tightly arranged. Compared with CON, COH had obvious neurofibropathy features, axon swollen, myelin sheath thickness increased, myelin lamellae loosened and disrupted, but complete demyelination and axonal dissolution did not yet occur. BAL also had a slightly axons and myelin lamina changes but to a lesser extent than COH (Fig. 10a). When the nerve myelin was significantly released, the myelin thickness was increased by lamellar separation, and G ratio (the diameter ratio of axon/axon plus myelin) (Fig. 10b) was significantly reduced, which is considered to be one of the manifestations of chronic degeneration of the optic nerve [19]. The G ratio in COH was significantly lower than that in CON and BAL, and the difference was statistically significant (*P* < 0.0001). However, there was no statistically significant different between CON and BAL, as shown in Fig. 10.

## Discussion

Glaucoma is an optic neuropathy, and its pathological mechanism is the loss and death of retinal ganglion cells due to the optic nerve axons damage. Previous studies have shown that increased intraocular pressure is a high-risk factor for glaucoma. However, recent studies have found that some patients’ disease continues to develop, although the IOP is under stable control [20]. As we known, glaucoma characteristic changes include thinning of RNFL, and loss of RGCs. Without an effective treatment, patients with chronic glaucoma are at high risk for visual field deficits, and eventually to complete vision loss.

TCM has a history of thousands of years of clinical practice and is widely used to treat glaucoma in China [21–23]. A meta-analysis performed by Li suggested that TCM interference could improve the visual acuity of glaucoma patients [24]. *Erigeron breviscapus, Radix salviae, Lycii fructus, Croci stigma* and *Ginkgo folium* are commonly used by practitioners of TCM in the treatment of chronic glaucoma [25–28], and their effects are believed to be related to improved eye microcirculation and increased blood flow [12, 29] and decreased oxidative stress [30], increasing the survival rates of RGCs and optic nerve axons [31, 32]. However, due to their complex composition and many targets, we cannot fully understand their mechanisms of action. Nevertheless, network pharmacology is a discipline that can predict the target of drug action and improve the efficiency of drug discovery, which can help in analyzing and understanding the targets and mechanisms of Chinese herbs [33]. Based on network pharmacology, we obtained 99 active ingredients and 146 target proteins related to the pathological progression of chronic glaucoma. However, most of the current studies of TCM have been limited to specific ingredient reactions with special targets, ignoring multiple ingredients and their cooperation in TCM. There are more herbal ingredients filtered by us that require further research to verify their therapeutic mechanism in glaucoma.

The PPI network constructed by common targets showed that many key proteins of biological pathways are involved in the herbal therapeutic processes in glaucoma, such as AKT1 (core protein of PI3K/AKT signaling), TP53 (a tumor suppressor gene coding tumor protein P53), STAT3 (core protein of JAK/STAT signaling), IL6 and IL1B (proinflammatory factors). Their interactions build complicated chain reactions in the process of glaucoma. Li’s research showed that PI3K/Akt signaling activation were positive correlation with the survival of RGCs both in vitro and in vivo in the simulated ocular hypertension environment, the activation of the PI3K/Akt pathway can inhibit RGC apoptosis in glaucoma [34]. An in vitro experimental study showed that IL6 activation regulates fibrosis of the trabecular meshwork induced by TGF-β to attenuate glaucoma progression [35]. Astrocytes, as the most widely distributed and abundant glial cells in the central nervous system, have also been confirmed to be involved in the pathological process of glaucoma. STAT3 plays a core role in the activation of astrocytes in glaucoma and can exhibit neuroprotective or neurotoxic effects on RGCs in different locations and conditions [36–38]. Since they are structural cells in the lamina, excessive proliferation of astrocytes can result in glial scarring and become an obstacle for regeneration of damaged axons of RGCs [39]. However, Danniel found that knockdown of STAT3 resulted in lower activated astrocytes and a higher apoptosis rate of RGCs in a rat glaucoma model [40]. Such a complicated PPI not only indicates the complexity of glaucoma but also suggests that the therapeutic effects of TCM involve multiple molecular mechanisms and network interactions of target proteins.

In the present GO biological process enrichment analysis, most of the important processes are included in our results, such as activation of transcription factors and activators, cytokine and related receptor activation, ubiquitylation and protease activity, messenger molecule activity and phosphatase activity. These processes manage cell DNA replication and transcription, functional protein synthesis and metabolism, signaling transmissions and so on.

As shown in KEGG enrichment analysis, multiple signaling pathways were involved in the treatment effects, such as the AGE-RAGE signaling pathway, IL-17 signaling pathway, HIF-1 signaling pathway, FoxO signaling pathway, and NF-κB signaling pathway, and the relevant mechanism included anti-neuroinflammation, antioxidative stress and glial activity regulation [41–45]. We performed molecular docking on the core target proteins of important signaling pathways, including AKT1 (1unq), BCL2 (2o22), HIF1α (4h6j), IL6 (4cni), MDM2 (5laz), STAT1 (3wwt) and STAT3 (6njs).

In this study, we established a chronic ocular hypertension model and found that BAL could significantly decrease IOP in this model (Fig. 7). These effects occurred possibly because BAL reduces Cl- and fluid secretion across the ciliary epithelium, thereby slowing the rate of aqueous inflow [46]. We also found that baicalein has a protective effect on glaucoma damage. The glaucoma model through EVC surgery caused COH group thickness of ganglion complex thinning and baicalein improved the change in BAL group (Fig. 8a/c). The COH group contained fewer RGCs in GCL compared with the CON group (Fig. 9a/b), and the BAL group contained more RGCs (Fig. 9a/b) and fewer significantly apoptotic cells in GCL than COH group. The Bcl-2 family is the key regulator in apoptosis and is closely associated with the loss of RGCs in glaucoma [47]. Our results indicated that baicalein can upregulate the expression of BCL-2 (Fig. 9c/d), consistent with the results of molecular docking. These protective effects of baicalein on glaucoma damage might be related to the activation of BCL-2 expression, decreasing of apoptotic cells in the GCL. The theoretically g-ratio in central nervous system is calculated to be around 0.7 [48, 49]. Compared with the CON group, the COH group g-ration decreased (Fig. 10c), and had axon swollen, an increased myelin sheath diameter and loose myelin sheath, while the BAL group g-ratio no significantly change compared with CON group (*P* > 0.05) (Fig. 10c), and axons were arranged tightly (Fig. 10a). Glaucoma involves primary injury of RGC axons and secondary degeneration of neural tissue, and the secondary loss of neural tissue in turn released more glutamate into the extracellular space caused excitotoxicity in nearby tissue. High levels of radical species may disrupt proteolipid protein and myelin protein through lipid peroxidation [50], leading to loose of myelin sheath and increase in thickness of myelin sheath [51]. This outcome shows that BAL has a protective effect on optic nerve may related to adjusted the level of extracellular glutamate [52]. Our network pharmacology research was based on the recent status of treatment, and the experiment on the mechanism of baicalein was not in depth, suggesting that future studies could increase in vitro experiments, perfect drug concentration screening, and block more comprehensive verification of theoretical predictions.

## Conclusion

In this study, we explored the protective effects and potential molecular mechanisms of TCM in the treatment of glaucoma through network pharmacology and used baicalein to treat an animal model of COH for observation. Network pharmacology analysis demonstrated that the mechanisms of herbal therapy against glaucoma were multicomponent, multitarget, and multipathway. Their interactions built complicated chain reactions in the process of glaucoma. Moreover, AKT1, TP53, STAT3, IL-6, IL-1b and several pathways, which are closely related to inflammation and oxidative stress, are believed to play a significant role in the mechanism of action of herbal therapeutics against glaucoma. Molecular docking results showed that the core active ingredients showed good affinity for BCL-2, IL-6, STAT3, HIF-1α, AKT1 and so on since the binding sites had stable hydrogen bonds. However, there is no in vivo evidence to support these interactions. Further studies are needed. Experimental evidence revealed that baicalein treatment notably decreased the IOP, reduced the axon and myelin sheath damage. The therapeutic effects of baicalein on glaucoma might be related to reduced apoptosis in GCL and up-regulated the expression of BCL-2 in a COH rat model, at least partially verifying the predicted consequences of network pharmacology. These findings provide direct evidence for TCM therapy in the prevention and treatment of glaucoma.

## Data Availability

The datasets used and/or analyzed during the current study are available from the corresponding author upon reasonable request.

## Abbreviations

ACG: Angle-closure glaucoma
ACA: Anterior chamber angle
A /APM: Axon/Axon plus Myelin
ARVO: Association for Research in Vision and Ophthalmology
AKT1: Threonine kinase 1
BAX: Bcl-2-associated X protein
BCL-2: B-cell lymphoma 2
BAL: Baicalein group
CNS: Central nervous system
CON: Control group
COH: Chronic Ocular Hypertension group
CMC-Na: Sodium carboxymethyl cellulose
DL: Drug likeness
DRTs: Drug-related targets
EVC: Episcleral veins cauterization
GO: Gene Ontology
GRTs: Glaucoma-related targets
GCC: Ganglion cell complex
GCL: Ganglion cell layer
H&E: Hematoxylin–eosin
HIF-1α: Hypoxia-inducible factor 1, alpha subunit
IOP: Intraocular pressure
IL-6: Interleukin 6
IL-17: Interleukin 17
Jak-STAT: Janus kinases-Signal transducer and activator of transcription
KEGG: Kyoto Encyclopedia of Genes and Genomes
MDM2: Mouse double minute 2 homolog
NF-κB: Nuclear factor kappa-B
NFL: Nerve fiber layer
OAG: Open-angle glaucoma
OB: Oral bioavailability
PPI: Protein–Protein Interaction
RGCs: Retinal ganglion cellsSD: Sprague–Dawley
STAT3: Signal transducer and activator of transcription 3
STAT1: Signal transducer and activator of transcription 1
TCM: Traditional Chinese Medicine
TCMSP: Traditional Chinese Medicine Systems Pharmacology
TUNEL: TdT-mediated dUTP Nick-End Labeling
TEM: Transmission electron microscopy
TP53: Tumor protein p53
TNF: Tumor necrosis factor
TGF-β: Transforming growth factor beta
Wnt: Wingless and Int-1
WHO: World Health Organization
